# Early Detection of Meningitis Outbreaks: Application of Limited-baseline Data

**Published:** 2017-10

**Authors:** Manoochehr KARAMI, Maryam GHALANDARI, Jalal POOROLAJAL, Javad FARADMAL

**Affiliations:** 1. Social Determinants of Health Research Center, Hamadan University of Medical Sciences, Hamadan, Iran; 2. Dept. of Epidemiology, School of Public Health, Hamadan University of Medical Sciences, Hamadan, Iran; 3. Research Center for Health Sciences, Hamadan University of Medical Sciences, Hamadan, Iran; 4. Modeling of Noncommunicable Diseases Research Center, Hamadan University of Medical Sciences, Hamadan, Iran

**Keywords:** Public health surveillance, Cumulative sum, Meningitis, Outbreak, Iran

## Abstract

**Background:**

There is no published study evaluating the performance of cumulative sum (CUSUM) algorithm on meningitis data with limited baseline period. This study aimed to evaluate the CUSUM performance in timely detection of 707 semi-synthetic outbreak days.

**Methods:**

Simulated outbreaks were generated using syndromic data on fever and neurological symptoms from Mar 2010 to Mar 2013 in Hamadan Province, the west of Iran. The performance of CUSUM algorithms, numbered from 1 to 11, in timely detection of outbreaks was measured using sensitivity, specificity, false alarm rate, likelihood ratios and area under the receiver operating characteristics (ROC) curve.

**Results:**

The highest amount of sensitivity was related to algorithm11 (CUSUM_(3–9 D11)_) and it was 52% (95% CI: 49%, 56%). Minimum amount of false alarm rate was related to CUSUM_(1–7 D5)_ algorithm equal to 8% (95% CI: 5, 10) and the best amount of positive likelihood ratio was related to CUSUM_(1–7 D4)_ equal to 4.97. CUSUM_(1–7 D1)_ has the best performance with AUC curve equal to 73% (95 CI%: 70%, 76%), as well.

**Conclusion:**

The used approach in this study can be the basis for applying CUSUM algorithm in conditions that there is no access to recorded baseline data about under surveillance diseases or health events.

## Introduction

Despite recent advances in the diagnosis of infectious diseases and the development of programs of vaccination against meningococcal meningitis, acute meningitis is still an important cause of illness and a serious threat to mankind and its related diseases annually causes 170,000 deaths in the world (1, 2). In Iran, meningitis is a notifiable disease and suspected cases of this disease i.e. syndromic data on fever and neurological symptoms are reported and registered daily in national surveillance system for communicable diseases (3).

Early detection and timely response to occurred outbreaks is a priority for public health authorities (4, 5). Generally, conventional surveillance systems respond to outbreaks based on laboratory time-consuming diagnosis. A new type of public health surveillance systems have special potential for rapid and timely detection of outbreak that known as syndromic surveillance system (6). Syndromic surveillance systems used different data sources and methods to detect outbreaks or unusual increases of health events (7, 8). One of the known algorithms for early detection of outbreaks is Cumulative Sum (CUSUM) algorithm, which is able to detect small changes in the process mean that control charts more quickly (9). CUSUM is under the umbrella of statistical process control-based methods (6), and it is used especially when historical data isn’t available or existing data are incorrect (10–12).

Outbreak detection algorithms methods typically require historical baseline data for a long period such as about 3–5 yr ago to be applied practically (13). Public health surveillance systems in order to identify outbreaks in shorter time as soon as possible have access to short term baseline data and less than three years (14). Consider to the lack of access to long period baseline data for majority of communicable diseases or newly emerging health events in many countries. It is necessary to develop applied algorithms for such situation.

The necessity of required limited baseline data algorithms is more tangible while staff of public health surveillance systems faced to similar circumstances like SARS epidemic.

Accordingly, this study aimed to evaluate the performance of CUSUM algorithm in timely detection of meningitis outbreak without long-term baseline data (Limited baseline data) based on semi synthesis approach in Hamadan Province.

## Methods

### Data

The performance of CUSUM algorithms was approached on syndromic data on fever and neurological symptoms (suspected cases of meningitis) from Mar 2010 to Mar 2013 in Hamadan Province, western Iran. We enrolled aggregate data of 1506 cases with sudden onset of fever above 38 °C and a clinical sign or symptoms such as neck stiffness, loss of consciousness, headache, vomiting and sudden neurological complications, reported daily in national surveillance system for communicable diseases in the province. To evaluate the performance of the algorithm, simulated outbreaks have been injected to pre-processed data of suspected cases of meningitis. Details on methodology of data preprocessing and removing explainable patterns have been described elsewhere (15).

### Outbreak Simulation

**Fig. 1:**
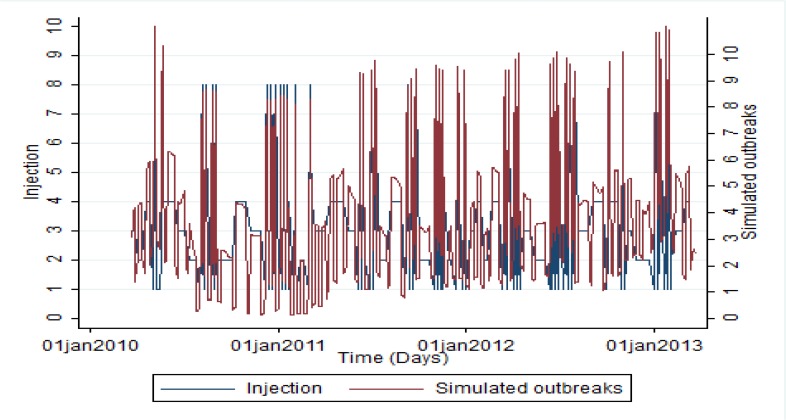
Size and duration of both injected cases on baseline cases and simulated outbreaks

Different types of meningitis related epidemic curves, consider to possible size, duration and shape, were generated. In case of size, we injected one to eight more cases to report suspected cases of meningitis during study period. The corresponding time duration of epidemic curve was 7, 14 and 21 d. Shapes of simulated outbreaks had uniform, exponential and linear distribution. Of 55 simulated epidemic curves, 23 outbreaks were 7 d period (15 uniform, 4 exponential and 4 linear), 18 outbreaks were 14 d period (10 uniform, 4 exponential and 4 linear) and other outbreaks were 21 d (8 uniform, 3 exponential and 3 linear). Finally, concerning epidemiological profile of meningitis and dynamics of disease transmission, 707 outbreak days were injected to 1085 d of real data on meningitis as known in literature as semi-synthetic outbreak. Accordingly, of 1085 d, 378 d were without outbreak. Fig. 1 depicts the size and duration of both injected cases on baseline cases and simulated outbreaks.

### Outbreak Detection Algorithm

We used CUSUM algorithms to detect outbreaks as follows. In this study, 11 CUSUM algorithms was evaluated based on limited data from the previous 7 d in closest proximity to the current value(days _t-1_ through _t-7_), or based on limited data from the past 7 d with an interval of two days, (days _t-3_ through _t-9_) with different threshold levels of meningitis outbreaks. The baseline period is always chosen from one week ago or each week attributed to the current value. CUSUM equation can be written as follows (14):

Equation 1: $${\text{CUSUM}}_{\text{t}}=\text{MAX}\;\left(0,{\text{CUSUM}}_{\text{t}-1}+{\text{Y}}_{\text{t}}-\sigma /2\right).$$

Where, Y_t_ is number of reported suspected cases of meningitis on day t (t = 1, 2... n), CUSUM_t-1_ is the value of CUSUM on day _t-1_ and σ is the standard deviation of observed data during the interesting week.

Totally, 11 different CUSUM algorithms with different characteristics, numbered from 1 (CUSUM_(1–7 D1)_) to 11 (CUSUM_(3–9 D11)_) as listed in Table 1, were studied and applied to 1085 semi-synthetic outbreak and non-outbreak days. The upper control limit or level of alarm threshold for CUSUM algorithm was calculated using limited baseline period based on a one sided positive CUSUM calculation. The corresponding formula is shown in Equation 2 .

**Table 1:** Characteristics of the used algorithms in the study according to fixed Parameter and time period

***Algorithm No.***	***Algorithm***	***Time period***	***h(Fixed Parameter)***
Algorithm 1	CUSUM_(1–7 D1)_	1–7 d ago	1
Algorithm 2	CUSUM_(1–7 D2)_	1–7 d ago	1.5
Algorithm 3	CUSUM_(1–7 D3)_	1–7 d ago	2
Algorithm 4	CUSUM_(1–7 D4)_	1–7 d ago	2.5
Algorithm 5	CUSUM_(1–7 D5)_	1–7 d ago	3
Algorithm 6	CUSUM_(3–9 D6)_	3–9 d ago	1
Algorithm 7	CUSUM_(3–9 D7)_	3–9 d ago	1.5
Algorithm 8	CUSUM_(3–9 D8)_	3–9 d ago	2
Algorithm 9	CUSUM_(3–9 D9)_	3–9 d ago	2.5
Algorithm 10	CUSUM_(3–9 D10)_	3–9 d ago	3
Algorithm 11	CUSUM_(3–9 D11)_	3–9 d ago	Three times deviation from mean of three past days

Equation 2: Upper Control Limit=UCL=μ+h×σ

Where μ is the mean of observed data during the interesting week and h is an appropriate value (fixed Parameter) ranges from 1 to 3 here. σ is the standard deviation. In algorithm 11, level of threshold is determined as three-time positive deviations from mean during three past days. CUSUM_t_ values are greater than the threshold level, algorithm alerts which indicate outbreak occurrence at given day.

### Measures of algorithm's performance

Indices of sensitivity, specificity, false alarm rate, false negative rate, positive and negative likelihood ratios used to report the performance of the CUSUM algorithms in timely detection of outbreaks timely. Total numbers of outbreak days (707 outbreak days) were considered as gold standard to calculate appropriate indices to evaluate performance of algorithms. Accordingly, the denominator for sensitivity and specificity formulas was 707 outbreak days and 378 non-outbreak days, respectively.

Area under Receiver Operating Characteristic (ROC) curve with 95% confidence intervals was used to compare different algorithms and greater values indicate better performance. Timeliness index of outbreak detection (time interval between the actual day of outbreak (Gold Standard) and the creation of alert by algorithm) was used to evaluate timeliness. The central and dispersion indices such as mean, standard deviation, and measures of performance except ROC were calculated using Microsoft Excel version 2010. ROC curve plotted using the Stata software version 11.

## Results

**Fig. 2:**
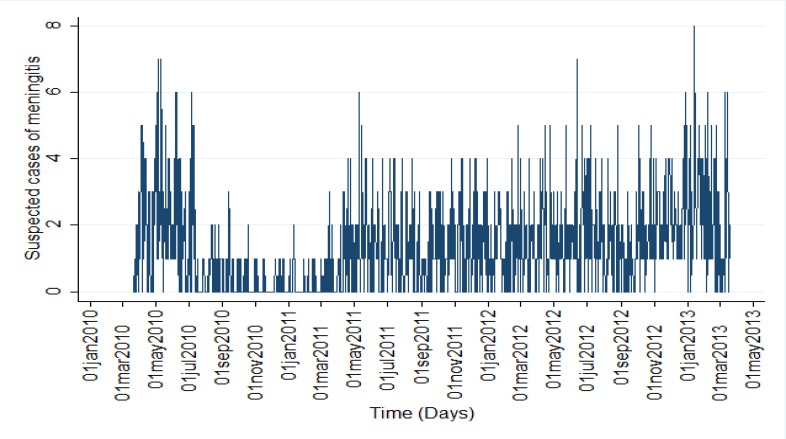
Line plot of suspected cases of meningitis from 21 March 2010 to 20 March 2013

During the study period of applied data, 1506 suspected cases of meningitis were reported and included as well. Details on descriptive statistics for fever and neurological symptom syndrome i.e. suspected cases of meningitis from Mar 2010 to Mar 2013 as depicted in Fig. 2 in Hamadan Province were described elsewhere (16, 17).

The highest amount of sensitivity was related to CUSUM_(3–9 D11)_ which equals to 52% (95% CI:49%, 56%). The highest value of specificity was related to CUSUM_(1–7 D5)_ and equals to 92% (95% CI:90%, 95%). The minimum amount of false alarm rate was related to CUSUM_(1–7 D5)_ and equals to 8% (95% CI: 5%, 10%) and minimum amount of false negative rate was related to CUSUM_(3–9 D11)_. The best amount of positive likelihood ratio was related to CUSUM_(1–7 D4)_ and equals to 4.97, and minimum negative likelihood ratio was related to CUSUM_(1–7 D1)_. More details on measures of algorithms' performance including sensitivity, specificity, false alarm rate, false negative rate, positive and negative likelihood ratios analyses are shown in Table 2.

**Table 2:** Sensitivity, specificity, false alarm rate, false negative rate, positive and negative likelihood ratios of the CUSUM algorithms

***Algorithm***	***Sensitivity***	***Specificity***	***False alarm rate***	***False negative rate***	***Positive Likelihood Ratio***	***Negative Likelihood Ratio***
CUSUM_(1–7 D1)_	45 (42–49)^a^	88 (85–92)	12 (8–15)	55 (51–58)	3.9	0.62
CUSUM_(1–7 D2)_	42 (38–46)	89 (86–96)	11 (7–14)	58 (54–62)	3.97	0.65
CUSUM_(1–7 D3)_	41 (37–45)	91 (88–94)	9 (6–12)	59 (55–63)	4.68	0.65
CUSUM_(1–7 D4)_	39 (36–43)	92 (89–95)	8 (5–11)	61 (57–64)	4.97	0.66
CUSUM_(1–7 D5)_	37 (33–40)	92 (90–95)	8 (5–10)	63 (60–67)	4.78	0.69
CUSUM_(3–9 D6)_	40 (37–44)	76 (72–80)	24 (20–28)	60 (56–63)	1.68	0.78
CUSUM_(3–9 D7)_	39 (36–43)	79 (75–83)	21 (17–25)	61 (57–64)	1.90	0.77
CUSUM_(3–9 D8)_	38 (34–41)	80 (76–84)	20 (16–24)	62 (59–66)	1.94	0.77
CUSUM_(3–9 D9)_	37 (33–40)	83 (79–87)	17 (13–21)	63 (60–67)	2.16	0.76
CUSUM_(3–9 D10)_	33 (30–37)	83 (79–87)	17 (13–21)	67 (63–70)	1.96	0.8
CUSUM_(3–9 D11)_	52 (49–56)	75 (71–80)	25 (20–29)	48 (44–51)	2.13	0.63

a Numbers in parenthesis indicate 95% confidence intervals around the point estimate

**Fig. 3:**
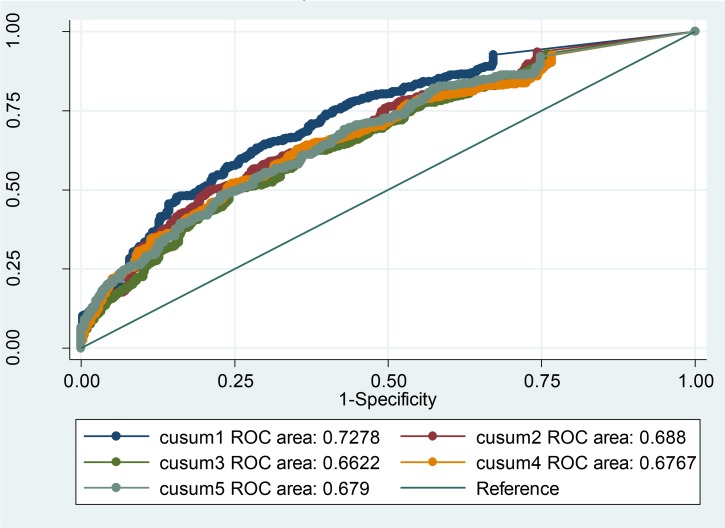
Area under the ROC curve for different CUSUM algorithms with baseline period 1–7 days by different parameters of h

**Fig. 4:**
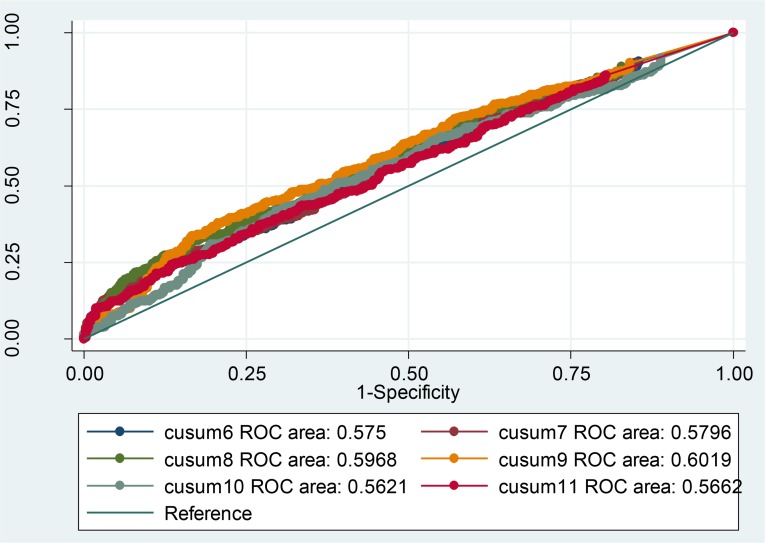
Area under the ROC curve for different CUSUM algorithms with baseline period 3–9 d

The CUSUM_(1–7 D 1),_ algorithm 1, with the ROC value 73% (95% CI: 70, 76) has shown the best performance in comparison with other algorithms (Fig. 3). Fig. 4 shows the ROC values for different CUSUM algorithms with baseline period during 3–9 past days.

Corresponding values for timeliness index according to shape of simulated outbreaks are shown in Table 3.

**Table 3:** Mean and standard deviation of timeliness index of the CUSUM algorithm to separate type of outbreak

***Algorithm***	***Timelines index for total outbreaks (Days)***		***Timelines index for simulated outbreaks (Days)***					
			***Uniform***		***Exponential***		***Liner***	
	***Mean***	***SD***	***Mean***	***SD***	***Mean***	***SD***	***Mean***	***SD***
CUSUM_(1–7 D1)_	7	5.58	6.93	4.32	5.67	3.89	6.23	4.37
CUSUM_(1–7 D2)_	7.1	5.63	7.01	4.26	5.3	3.8	6.3	4.3
CUSUM_(1–7 D3)_	7.08	5.54	7.06	4.29	5.3	3.8	6.08	4.34
CUSUM_(1–7 D4)_	7.02	5.57	6.96	4.24	5.2	3.92	6.2	4.5
CUSUM_(1–7 D5)_	7.17	5.56	7.41	4.39	5.19	3.82	6.01	4.48
CUSUM_(3–9 D6)_	6.18	5.2	6.2	3.87	4.8	3.5	5.02	3.6
CUSUM_(3–9 D7)_	6.27	5.22	6.35	3.93	4.6	3.53	5.2	3.8
CUSUM_(3–9 D8)_	6.37	5.26	6.44	3.96	4.6	3.6	5.3	4.09
CUSUM_(3–9 D9)_	6.35	5.25	6.47	4.04	4.3	3.29	5.2	4.1
CUSUM_(3–9 D10)_	6.52	5.33	6.6	3.94	4.52	3.53	4.75	3.67
CUSUM_(3–9 D11)_	5.94	4.82	5.79	3.75	5.3	3.66	5.55	3.85

Minimum and maximum time interval between the actual day of outbreak and the time of alarm by algorithm were observed in CUSUM_(3–9 D11)_ with 5.94 d and in CUSUM_(1–7 D5)_ with 7.17 d, on average. Moreover, simulated outbreaks with exponential distribution have worked timely in comparison with uniform and linear distributions. So the best performance in timely detection of outbreaks was related to CUSUM_(3–9 D9)_.

## Discussion

Since any algorithm has not ability to detect all outbreaks effectively and evaluate the performance of algorithms provides useful and important information regarding strengths and weaknesses of applied algorithm, this study was designed to evaluate the performance of CUSUM algorithms with limited baseline period and to identify timely detection of meningitis.

The results of this study as well as some studies aimed to evaluate the performance of the CUSUM algorithm in different circumstances, using semi synthesis and synthesis approach has good performance in detecting semi-synthetic outbreaks (18). Our findings are consistent with the results of another study (19), was aimed to evaluate the CUSUM performance to detect small deviations from the mean. The best performance in terms of sensitivity was observed for CUSUM_(3–9 D11)_ with an average data on 3–9 d ago. This result is consistent with the results of Hut Wagner et al. (14). However, our findings regarding false positive rate are in contrast to related study (14). This disagreement could be justified while considering the role data pre-processing in our study instead of raw data with explainable patterns.

Simulated data with regard to comprehensive characterization of the properties of outbreaks provides reliable information to compare outbreak detection methods. Application of simulated outbreaks with different size, shape and time periods and adding simulated outbreaks to preprocessed data of suspected cases of meningitis are main strengths of this work. Strength of this study is application of preprocessed values of LOWESS method (Details are described elsewhere as mentioned in methods) instead of the raw of suspected cases of meningitis, which reduces the false alarm rate, increase sensitivity, and timely detection of outbreak occurs.

The main limitations of the present study are (a) simulated outbreaks have been generated consider to knowledge of the dynamics of meningitis in Hamadan Province and it is different from other data, thus it is limited to compare its results with other studies. (b) Applied algorithms are evaluated under near real conditions in the era of possible outbreaks, not real conditions. To resolve this problem we state comprehensive information about added outbreaks, that including size, shape and period of potential outbreaks. However, we have not accessed to official data regarding any outbreak occurred during study period to evaluate algorithms using actual data source as gold standard (4).

## Conclusion

The used approach in this study could be the basis for applying CUSUM algorithm in conditions that there is no access to recorded baseline data on interested diseases or health events. Nevertheless, the simultaneous using of other methods of outbreak detection is recommended. Methodology of this work is not limited to meningitis and can be applied to other syndromic data in public health surveillance.

## Ethical considerations

Ethical issues (Including plagiarism, informed consent, misconduct, data fabrication and/or falsification, double publication and/or submission, redundancy, etc.) have been completely observed by the authors.
